# Adsorptive treatment of brewery effluent using activated *Chrysophyllum albidium* seed shell carbon

**DOI:** 10.1186/2193-1801-3-213

**Published:** 2014-04-30

**Authors:** Matthew Chukwudi Menkiti, Mathew Chidiebere Aneke, Paul Madus Ejikeme, Okechukwu Dominic Onukwuli, Nwasinachi Uzoma Menkiti

**Affiliations:** Department of Chemical Engineering, Nnamdi Azikiwe University, Awka, Nigeria; Department of Process Engineering, Stellenbosch University, Stellenbosch, South Africa; Department of Pure and Industrial Chemistry, University of Nigeria, Nsukka, Nigeria; Department of Chemistry, University of Pretoria, Pretoria, 0002 South Africa; Center for Environmental Management and Control, University of Nigeria, Enugu Campus, Enugu, Nigeria

**Keywords:** Brewery effluent, *Chrysophyllum albidium*, Adsorption isotherms, Suspended and dissolved particles

## Abstract

*Chrysophyllum albidium* seed shell, an abundant, biodegradable and inexpensive natural resource was used as a precursor to bioadsorbent production for the removal of suspended and dissolved particles (SDP) from initially coagulated Brewery Effluent (BRE). Influence of key parameters such as contact time, bioadsorbent dose, pH and temperature were investigated using batch mode. The thermal behavior studies were evaluated using Thermogravimetric and Differential scanning calorimetric analyses. The morphological observations and functional groups of the bioadsorbents were determined using scanning electron microscopy and Fourier transform infrared spectroscopy, respectively. The adsorption equilibrium, thermodynamics and kinetic of SDP adsorption on H_3_PO_4_-treated shell and NH_4_Cl-treated shell were examined at specified temperatures. Equilibrium data sufficiently fitted the Langmuir isotherm model (*R*^2^ > 0.99; *SSE* < 0.09). The pseudo-second order kinetic model provided the best correlation (*R*^2^ > 0.99; *SSE* < 0.14) with the experimental data. The values of *ΔG°* and *ΔH°* indicated the spontaneous and endothermic nature of the process. This study demonstrated that *C. albidium* seed shell could be utilized as low cost, renewable, ecofriendly bioadsorbent for the uptake of SDP from BRE.

## Introduction

Brewing is an intensive water consuming activity, besides utilizing a wide variety of chemicals. Expectedly, large volumes of effluent is discharged into water courses of brewery bearing communities, leaving in its wake a polluted aquifer (Khuo-Omoregbe et al. 2005; Menkiti 2010). Increasing concentration of these organic/non-organic enriched BRE in the water constitute a severe health hazard to both plants and animals, thus impeding the functionality of the ecosystem. The situation is typical of the BRE receiving aquatic system in Nigeria, where much of the water resources cannot be utilized without a form of treatment, following effluent discharges with negligible consideration for environmental control (Menkiti and Onukwuli 2011a).

BRE, generated from lager beer production, contains large amount of SDP (Menkiti and Onukwuli 2010). Typically, the organic contents of BRE consists of sugars, soluble starch, ethanol, volatile fatty acids and solids which are mainly spent grains, yeast and trub (Driessen and Vereijken 2003). Untreated BRE quantitatively contains suspended solids (100–1500 mg/l), chemical oxygen demand (300–800 mg/l), nitrogen (30–100 mg/l) and phosphorus (10–30 mg/l) (Menkiti *et al.*2011a World Bank Group 1998).

Over the years, significant attention has been given to the environmental cleanup of such contaminated aqua system using varieties of techniques such as precipitation, ion-exchange, coagulation, reverse osmosis and adsorption (Hameed and El-Khaiary 2008; Larous *et al.*^2005^; Menkiti *et al.*2011b; Menkiti and Onukwuli 2011b). Among the different treatments listed above, adsorption technology is attractive due to its merits of efficiency,even at low concentration of contaminants (Meena et al. 2008; Yakubu et al. 2008), economy, simple operation and insensitivity to toxic substances (Grini 2005; Feng *et al.*^2010^; Menkiti and Onukwuli 2011b).

The common adsorbents primarily include zeolite, clays, polymeric materials and natural agricultural materials which provided the focus of this study (Asasian and Kaghazchi 2013; Jiang *et al.*^2012^; Amirnia *et al.*^2012^). These natural materials have potential to be used as low cost bioadsorbent, as they represent unused resources, abundantly available and known to be eco-friendly (Deans and Dioxn 1992). Progressively, much attention has been focused on techniques of converting these waste materials into useful adsorbents. Among these agricultural wastes are saw dust (Meena et al. 2008) palm ash (Ahmad *et al.*^2007^).

*Chrysophylum albidium* seed shell, considered in this study, is of tropical forest tree of genus *chrysophyllum*, family of *sapotaca*e and order of *ericales.* The fruit is large berry containing 4 to 5 flattened seeds with a hard shell (Bada 1997). Significant quantities of these seeds shells are produced annually in Nigeria without being put to useful ends industrially. However, successful application of adsorbent from *C. albidium* seed shells for the removal of heavy metals had been reported (Oboh *et al.*^2009^; Onwu and Ogah 2010; Ejikeme *et al.*^2011^). According to the author’s knowledge, no attempt has been made until now to use this seed shell for the treatment of natural organic aqueous waste, such as BRE. Therefore, it was of interest to experiment with such a promising biomaterial for the adsorptive removal of SDP from initially coagulated BRE by batch technique. The work further seeks to investigate the influence of contact time, adsorbent dosage, temperature and effluent pH on the adsorptive uptake of SDP from the BRE. Also, the research investigated the ability of three isotherm models: namely the Langmuir, the Freundlich and the Temkin adsorption isotherms to model the equilibrium adsorption data. Another major focus of the work was the kinetic study, conducted to determine the rate of SDP adsorption and evaluation of which of the four kinetic models (pseudo-first order, pseudo-second order, Elovich and Bhattacharya-Venkobachar) that best describes the adsorption process. Finally, material characterization and thermodynamic analyses were conducted to present the characteristic properties and energy changes associated with the adsorption study.

## Materials and methods

### Materials collection

#### Brewery effluent

Brewery effluent was obtained from a beer brewery at 9^th^ Mile Corner Udi, Enugu Sate Nigeria and stored in black plastic container to preserve and further prevent changes in the characteristics of the effluent (Clesceri *et al.*^1999^).

#### *C. Albidium* seed shells

The precursor for the preparation of the bioadsorbents, *C. albidium* seed shells was obtained as a waste material from Nsugbe, Anambra State of Nigeria. Pretreatment of the shells by thorough washing with distilled water to remove the impurities was done and the washed sample dried in an air circulating oven at 40°C for 14 hours. The shell samples were blended, sieved and particles that ranged between 2 and 3 mm were obtained and stored in a desiccator for use in the experiments.

Two portions of the shell samples, of known weights, were immersed in 60% solutions of ammonium chloride and phosphoric acid, respectively, for 24 hours. The carbonization of the shell samples were carried out in a muffle furnace at 600°C for 4 hours, washed with distilled water to pH 7, dried at 110°C for 8 hours and subsequently sieved to desired diameter using standard sieves.

### Materials characterization

#### Brewery effluent

Standard APHA methods, as reported by Clesceri et al. (1999), were applied to determine the physiochemical and biological characteristics of the effluent.

#### Bioadsorbent

The physical and chemical characterization of H_3_PO_4_-treated *C. albidium* sawdust carbon (ASAA) and NH_4_Cl-treated *C. albidium* sawdust carbon (ASAS), shown in Table 1, was performed using the methods described in 2.2.2.1 to 2.2.2.6.

**Table 1:** **Characteristics of ASAA and ASAS**

Parameters	ASAA	ASAS
BET Surface area (m^2^/g)	273.832	137.289
Total pore volume (cm^3^)	2.8810	2.100
Bulk density (g/cm^3^)	0.4777	0.5905
% Ash content	2.513	1.701
Oil content (%)	0.9651	0.9621
Moisture content (%)	2.741	2.742

##### Tapped bulk density

This property was determined according to the methods of Ortega-Rivas (2012). A given weight *(w* g) of the bioadsorbent sample which was dried at 110°C was put in 10 ml measuring cylinder. The bottom of the cylinder was tapped gently on the laboratory bench top until there was no further change in the sample level. The bulk density was then calculated using Eq. 1:1

where: *w* is weight of the dry material and *v* is volume of the dry material.

##### Percentage ash content

The determination of percent ash content of the samples were done by putting exactly 3 g each of the pre-dried bioadsorbent sample into pre-weighed crucibles and subsequently burnt in a muffle furnace at 650°C for 5 hours in the presence of air. Average value of three determinations was recorded and the percentage ash content was calculated using Eq. 2.2

where: *W*_ash_ is weight of ash and *W*_sample_ is weight of bioadsorbent sample.

##### Percentage moisture content

Exactly 3 g of the *C. albidium* seed shell (activated/carbonized) samples were weighed into pre-weighed crucible and the total weight taken. The samples were burnt at 105°C for 5 hours, removed from the oven, cooled and repeatedly heated and weighed after 1 hour until a constant weight was obtained. The % moisture content was determined using Eq. 3.3

where: *W*_sample_ is the weight of bioadsorbent sample before drying, *W*_dry_ is weight of sample after drying.

##### Pore volume

A 3 g each of the bioadsorbent was weighed out. The samples were completely immersed in water and boiled until the air in the bioadsorbents had been displaced. The samples were then superficially dried and weighed. The pore volume was calculated using Eq. 4.4

where: V_pore_ is pore volume of the bioadsorbent and *W*_*inc*_ is weight increase of the bioadsorbent and *ρ*_*water*_ is the density of water.

##### Surface area

Surface area was determined according to the modified methods of Kang et al. 2013. It was calculated from nitrogen gas adsorption isotherms based on Brunauer, Emmet and Teller (BET) method of surface area analysis (at 77.305 K) using Quantachrome 2.0 analyzer.

##### Physiochemical and instrumental characterization of bioadsorbent samples

Fourier transfer infrared analysis was carried out using Shimadzu FTIR 8400S spectrophotometer for the determination of functional groups present in ASAA and ASAS. The thermal behavior of the activated carbon was evaluated by thermogravimetric and differential scanning calorimetric analyses using TGA-Q 50 and DSC-Q 200 models, respectively. The surface morphology of the activated carbon was visualized via scanning electron microscopy (SEM) using a scanning electroscope model FEI-QUANTA 200. Physiochemical and instrumental characterization of the bioadsorbents were conducted using standard methods (Feng et al. 2010).

### Adsorption experiments

Batch adsorption experiments were performed in Erlenmeyer flask according to the methods reported by Sivakumar and Palanisamy (2009) and Yeddou and Bensmaili (2005). In a typical experiment, 20 ml of effluent sample was mixed with the appropriate amount of adsorbents (types ASAA and ASAS) in the range of 10-50 g/l and then shaken for a period of time ranging from 5 to 60 min at 20 rev/s. The temperatures of adsorption measurements ranged between 20 and 30°C and the appropriate value was applied accordingly as required. The sample was then filtered using Whatman no 42 filter paper having fine porosity and particle retention of 2.5 micrometer at slow flow rate in a glass funnel. The filtered liquid was analyzed with respect to particle (SDP) content.

### Analytical method

Filtrate from adsorbed BRE samples were taken at specified time range of 5–60 min, and then analyzed using a Spectronic (Milton Roy Company) 21 UV-visible spectrophotometer. The maximum uncertainty of the analytical methods was estimated at 5% level. The adsorption capacity, *q*_*t*_ (mg/g), and the percentage SDP removal, % Rem, were calculated using Eqs. 5 and 6, respectively.56

where: *C*_*0*_, *C*_*t*_ and *D*_*A*_ are initial constant effluent concentration (mg/l), effluent concentration at any time, t and adsorbent dose (g/l), respectively.

## Results and discussion

### Characterization of results

#### Physiochemical and biological characteristics

The results of the physiochemical and biological characteristics of the coagulated BRE, along with the regulatory standard (FEPA-Federal Environmental Protection Agency 1991), are presented in Table 2. The major characteristics (turbidity, total dissolved solid, total suspended solid, biological oxygen demand) contributing to the cloudiness of the fluid were relatively in low concentrations, but significant enough to promote adsorption process in the medium. Usually low concentration of contaminant is one of the key conditions that favor the application of adsorption (Meena et al. 2008; Yakubu et al. 2008). Meanwhile, the characteristics of adsorbents presented in Table 1 showed that ASAA had larger surface area/pore volume than the ASAS, an indication of likely better adsorptive performance of the former.

***Table 2:** **Characterization result of Brewery Effluent and FEPA standard**

Parameters	Value	FEPA Limit
pH	2.4400	6-9
Turbidity (NTU)	39.0000	-
Total hardness (mg/l)	150.0000	-
Ca^2+^(mg/l)	47.2000	-
Mg^2+^(mg/l)	12.6000	-
Fe^2+^(mg/l)	0.1000	-
SO_4_^2-^(mg/l)	65.2500	-
NO_3_^2-^(mg/l)	0.1500	20
Cl^−^(mg/l)	614.8089	<1
E.cond (μm/m^2^)	158.7000	400-800
TDS (mg/l)	8.8870	<2000
TSS (mg/l)	2.0900	15-30
T.Coliform	Nil	-
Plate Count	1.0000	-
E-Coli	Nil	-
BOD_3_	6.1405	10-50

*Based on standard APHA method: Clesceri et al. 1999.

#### FTIR spectra

**Figure 1:**
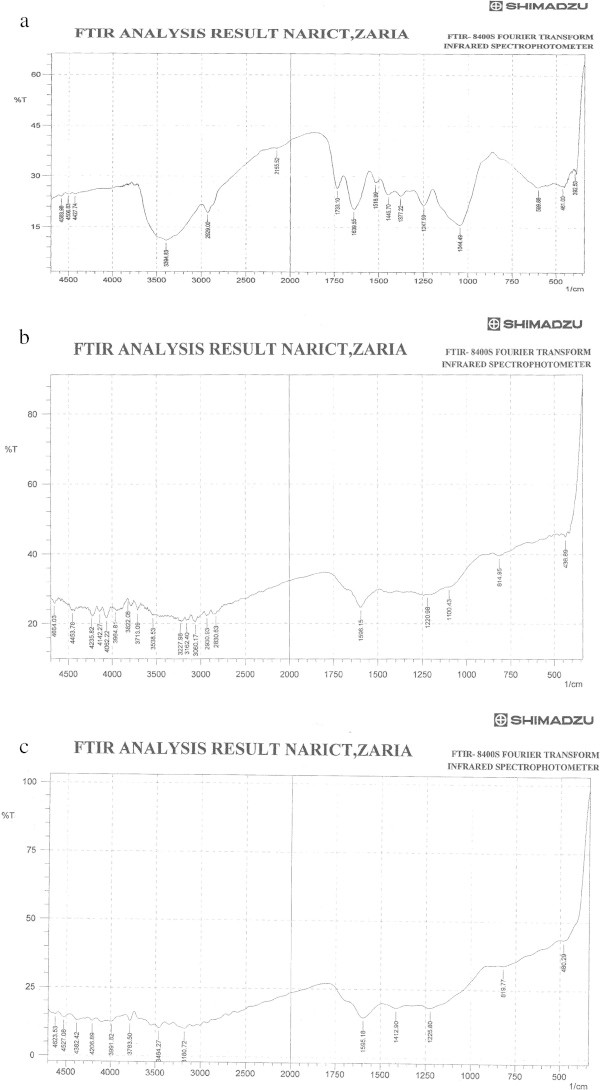
**FTIR Spectra of (a): Precursor sample (UASS) (b): ASAA (c): ASAS.**

The IR transmittance of UASS, ASAA and ASAS plotted against wave number is displayed in Figure 1. From the region of the transmittance peaks and notional structure of the UASS, ASAA and ASAS, it was possible to assign some of the functional groups. The FTIR spectrum of UASS shown in Figure 1a indicated distinct peaks at 3394.83 cm^−1^ (O-H stretch), 2929 cm^−1^(CH stretch shift), 2155.52 cm^−1^(C ≡ C stretch shift), 1733.1, 1639.55 cm^−1^(C = O stretch), 1518.99 cm^−1^(NO_2_ asymmetrical stretch), 1445.7-1377.2 cm^−1^(C-H scissoring and bending), 1247.99-1044.49 cm^−1^(C-N stretch). Noted was the possibility of existence of aromatic rings from the peaks observed between 1550 and 1600 cm^−1^; though N-H bonding, for example, from –NH_2_ and NH_3_^+^ moieties, has an absorption band in this region. Also, the stretching frequencies of N-H bonds could also be confused with those of O-H frequencies in the 3100 and 3600 cm^−1^ region. In addition, the broad bands between 2700 cm^−1^ and 2250 cm^−1^ are characteristics of amine groups in solid phase (>NH_2_^+^, etc.).

The FTIR spectra of ASAA and ASAS are depicted in Figure 1b and c, respectively. For both figures, discernable peaks of note were recorded at 3991.82-3162.40 cm^−1^(O-H stretch/phenols), 3456.55 cm^−1^(O-H broad stretch/N-H medium stretch), 3060.17-2830.63 cm^−1^(O-H broad stretch), 1596.15-1595.16 cm^−1^(NO_2_ asymmetrical stretch), 1412.90 cm^−1^(C-H scissoring/bending), 1225.80 cm^−1^(C – N stretch/C-O stretch), 1220.98 cm^−1^(C-H/C-O stretches), 1100.43 cm^−1^(C-N/C-O stretches) and 814.95-819.77 cm^−1^ (C-H stretch bend).The FTIR spectra of used adsorbents showed (Figures are not shown) discernable peaks (in cm^−1^) for used ASAA at 461(S-stretching), 803(C-H stretch/NH_2_ wagging and twisting), 1084.03 (second overtone N-H and O-H stretching), 1419.66 (first overtone N-H and O-H stretching), 1608.69(pyridine C-N),3443.05 (primary NH_2_ asymmetric stretching), and for used ASAA at 389 (aliphatic P compound), 469 (S-stretching), 880.53/1101.39(aromatic P-O stretching), 1450.52/1573.97(C = C stretching), 3458.48(primary NH_2_),3633.05(O-H stretching).

The FTIR results of bioadsorbent precursor (Figure 1a), ASAA (Figure 1b), ASAS (Figure 1c) and used adsorbents indicated that some peaks were shifted or disappeared, and that new peaks were also detected. These changes observed in the spectra represent interactive effects due to involvement of those functional groups during production and use of the adsorbents. The changes observed in the peaks of the adsorbents after usage could be concluded to be the direct result of adsorptive uptake of SDP from the BRE by the adsorbents. The varying number of absorption peaks displayed by the samples reflected the complex nature of these materials (Hameed and El-Khaiary 2008; Chemistry Department at Colorado University CDCU 2002; Graham *et al.*^2008^).

#### DSC and TGA

**Figure 2:**
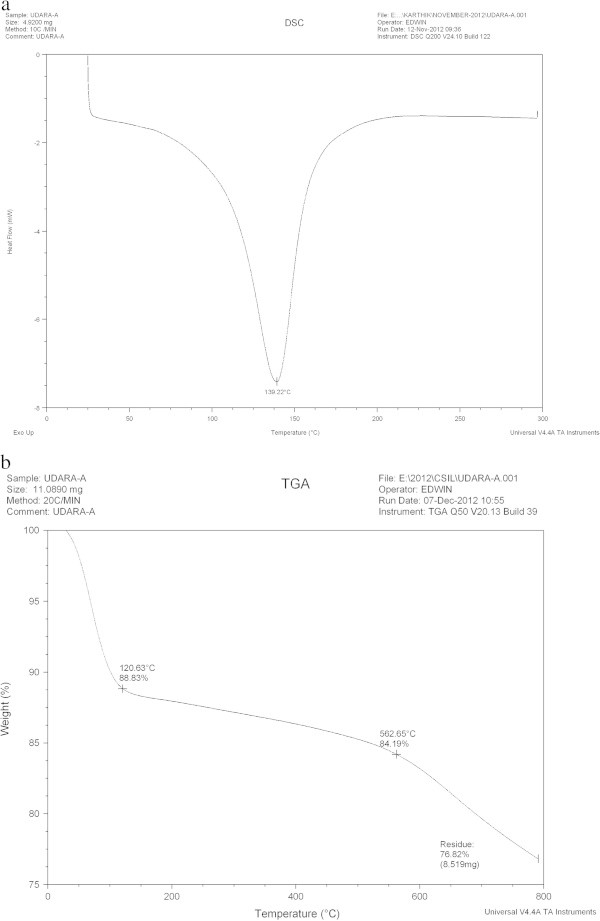
**Graph of: (a) DSC and (b) TGA of ASAA.**

**Figure 3:**
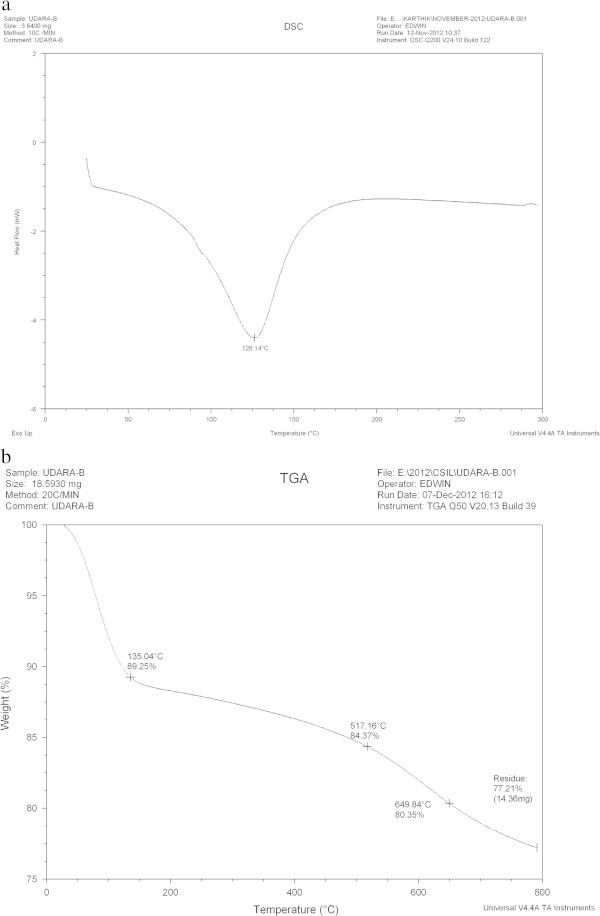
**Graph of: (a) DSC and (b) TGA of ASAS.**

The differential scanning calorimetry (DSC) and Thermo gravimetric analysis (TGA) curves for ASAA and ASAS are presented in Figures 2 and 3, respectively. DSC is a thermo analysis in which the difference in the amount of heat required to increase the temperature of a sample to that of reference is measured as a function of temperature. By observing the difference in heat flow between the samples and reference, DSC was able to measure the amount of heat absorbed or released during phase transition (Gill *et al.*^2010^). TGA is based on mass measurement of mass loss of material as a function of temperature. The loss of weight could result from chemical reaction (decomposition, combustion) and physitransition (evaporation, desorption, drying) (Vyazovkin 2012)

As indicated in Figures 2a and 3a, DSC was used to characterize the phase transition that occurred in ASAA and ASAS over the temperature range of 25-277°C. Figure 2a revealed a sharp thermal transition in the temperature range of 125-150°C with transition enthalpy of 250 kJ/kg. Figure 3a indicated sharp transition in the temperature range of 112.5-138°C with transition enthalpy of 819.9 kJ/kg. This behavior by both ASAA and ASAS could be attributed to the de-stringing and coiling of carbon chain leading to spontaneous densification (Ramani *et al.*^2012^). The densification of the aggregated mass occurred at 175-180°C and 150-175°C for ASAA (Figure 2a) and ASAS (Figure 3a), respectively without absorption of thermal energy. In other words, the heat flow discs indicated exothermicity.

The thermal decomposition behaviors of the bioadsorbents are illustrated in Figures 2b and 3b for ASAA and ASAS, respectively. Figure 2b shows that ASAA lost weight by 11.17% and 15.18% at 120.63°C and 562.65°C, respectively. For Figure 3b, the weight loss of 10.75, 15.63 and 19.65% were recorded for 135.04, 517.16 and 649.84°C, respectively. For both Figures 2b and 3b, the initial weight loss could be attributed to the internal moisture and gaseous loss from the matrix molecules of the adsorbents (Ramani *et al.*^2012^). The second phase weight loss may be attributed to the decomposition of the labile component in the adsorbent. The results presented in Figures 2 and 3 conclusively suggested operational stability of the adsorbents.

#### SEM image

**Figure 4:**
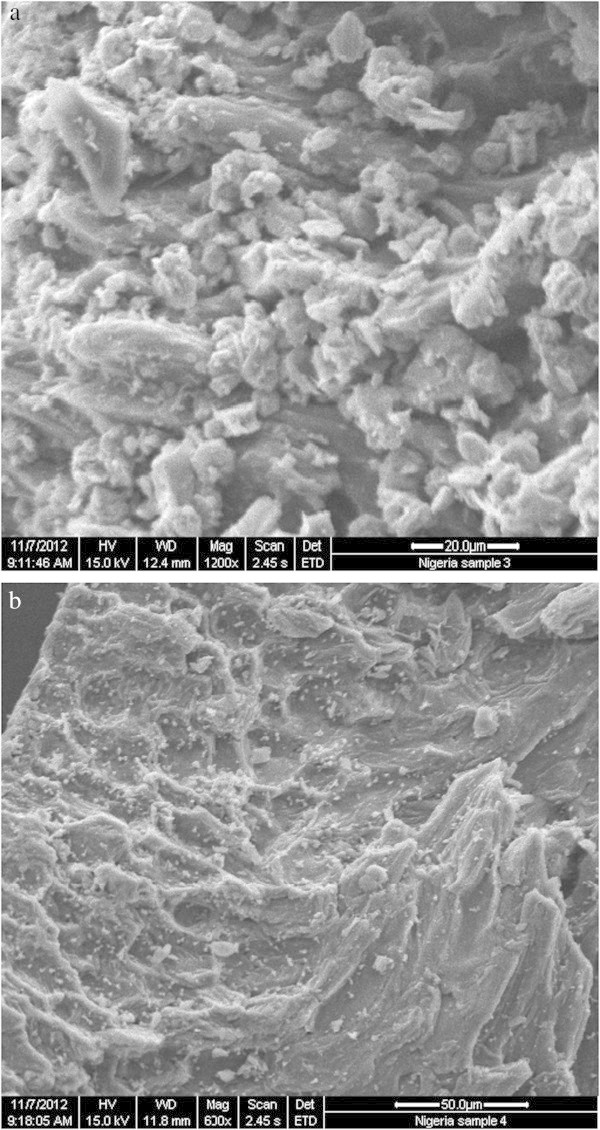
**SEM images of (a) ASAA (b) ASAS.**

Scanning electron micrographs of ASAA and ASAS are shown in Figure 4a and b, respectively. The micrographs in Figure 4 appear to be dark field, with illuminations dotted in the matrix. Both figures seem to be rough, with protrusions quite prevalent in the biomass. High level of porosity was observed on both ASAA and ASAS. However, the ASAA were relatively more porous than the ASAS. In addition, higher levels of blunted sponge-like protrusions (Kang *et al.*^2013^) were observed in ASAA when compared to ASAS. This was probable reason for relative better performance of ASAA when compared to ASAS. The more heterogeneous pores in ASAA ensured good possibility for the SDP to be trapped and adsorbed. The BET surface area values for the ASAA supported the apparent more heterogeneous porosity in ASAA. The BET surface areas for ASAA and ASAS were 273.832 and 137.289 m^2^/g, respectively.

### Influence of contact time and adsorbent dosage on adsorptive removal of SDP from BRE

**Figure 5:**
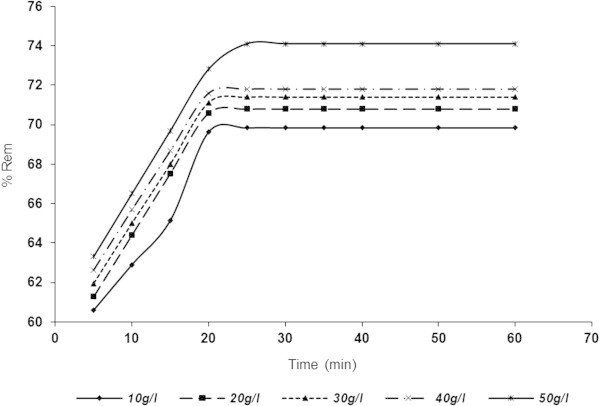
**Influence of contact time and varying ASAA dose on the % removal of SDP from BRE (BRE volume =20 ml, C**_**0**_ **= 161.92 mg/l, initial BRE pH, original temperature = 25°C).**

**Figure 6:**
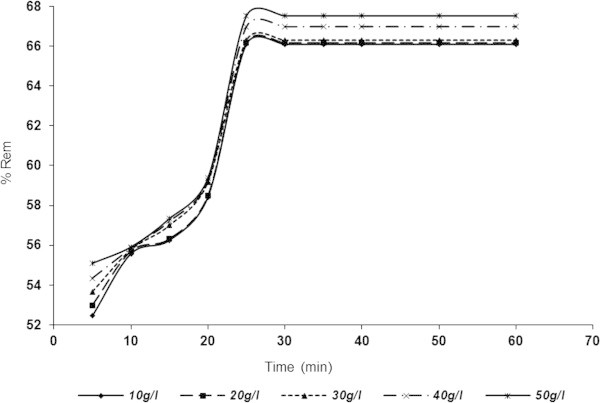
**Influence of contact time and varying ASAS dose on the % removal of SDP from BRE (BRE volume =20 ml, C**_**0**_ **= 161.92 mg/l, initial BRE pH, original temperature = 25°C).**

**Figure 7:**
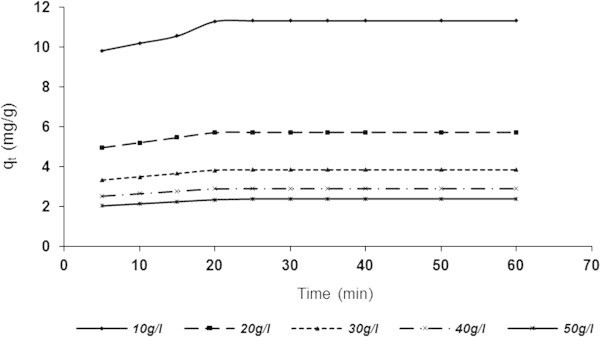
**Influence of contact time and varying ASAA dose on the adsorptive capacity (BRE volume =20 ml, C**_**0**_ **= 161.92 mg/l, initial BRE pH, original temperature = 25°C).**

**Figure 8:**
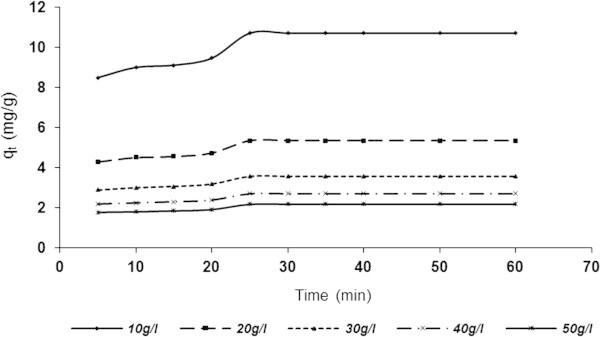
**Influence of contact time and varying ASAAS dose on the adsorptive capacity (BRE volume =20 ml, C**_**0**_ **= 161.92 mg/l, initial BRE pH, original temperature = 25°C.**

The results for adsorptive removal of SDP with respect to time and adsorbent doses are shown in Figures 5, 6, 7 and 8 over the range of 10–50 g/l. Figures 5 and 6 indicated that SDP removal increased with increase in adsorbent dosage. From the profiles of the Figs., the particle retention increased rapidly and tended towards constant after equilibrium time of 30 min for both ASAA (Figure 5) and ASAS (Figure 6). From Figure 5, the percentage removal recorded for ASAA at 10 g/l were 60.5978 and 69.8369% for the time of 5 and (30–60) min, respectively. For ASAA (Figure 5) at 50 g/l, 63.3152 and 74.1168% were recorded for 5 and (30–60) min, respectively. Similar trends were depicted in Figure 6 (ASAS) where 52.4456 and 66.1005% were achieved for time of 5 and (30–60) min, respectively at 10 g/l dose. For 50 g/l ASAS dose, 55.095 and 67.527% removal were achieved at 5 and (30–60) min, respectively. The results demonstrated that ASAA performed better than the ASAS at the conditions of this experiment.

The observed adsorption dynamic profiles depicted by Figures 5 and 6 can be divided into three regimes (Ncibi *et al.*^2008^): (i) a linear increase in adsorption with time, (ii) a transition regime where the rate of adsorption levels off, and (iii) a plateau regime. First regime (initial steep slope) indicated instantaneous adsorption (overshoot phenomena) ability of effluent particles onto the surface of the adsorbents. The second regime indicated a phase of gradual attainment of equilibrium where the apparent fall in SDP adsorption rate might be due to utilization of active sites on adsorbents surface. Plateau regime indicated phase where equilibrium had been achieved (Mohan *et al.*^2007^).

One general phenomenon was the perceived increase in SDP uptake with increase in adsorbent dose. It should also be observed that the % Rem for doses considered in Figures 5 and 6 ranged closely; with only apparent difference noticed for 50 g/l (Figure 5). This could be attributed to the greater availability of close number of exchangeable sites or surfaces in the adsorbents (Meena *et al.*^2008^). Following optimal results obtained at 50 g/l and 30 min, the rest of the work was carried out at the stated results, unless otherwise indicated.

Figures 7 and 8, present results similar to Figures 5 and 6 in respect of the variation of adsorption capacity *q*_*t*_ (mg/g) with contact time and adsorbent dose. It was evident that *q*_*t*_ increases with decreasing adsorbent dose and increasing contact time before leveling-off. The highest and lowest *q*_*t*_ were recorded at 10 and 50 g/l, respectively for both ASAA (Figure 7) and ASAS (Figure 8). In specific terms, *q*_*t*_ at 10 g/l increased from 9.812 to11.308 mg/g for 5 and (30–60) min, respectively as shown in Figure 7. Also, the *q*_*t*_ at 50 g/l ASAA increased from 2.0504 to 2.4002 mg/g for 5 and (30–60) min, respectively. Figure 8 indicated that q_t_ at 10 mg/l recorded increment from 8.4920 to 10.703 mg/g for 5 and (30–60) min, respectively while that of 50 g/l increased from 1.7842 to 2.1868 mg/g for 5 and (30–60) min, respectively. The results indicated also that the ASAA performed better than ASAS.

The apparent decrease in adsorption density (amount adsorbed per unit mass of the adsorbent) with increase in adsorbent dose was due to progressive unsaturation of adsorption sites through the adsorption reaction. Another reason might be due to the particle interaction, such as aggregation, usually resulting from high adsorbent concentration. Such aggregation would lead to decrease in total surface area of the adsorbent and on increase in the diffusional path length (Shukla *et al.*^2002^). Particle interaction might also desorb some of the adsorbate that was only loosely and reversibly bound to the adsorbent surface.

### Influence of temperature on the adsorptive removal of SDP from BRE

**Figure 9:**
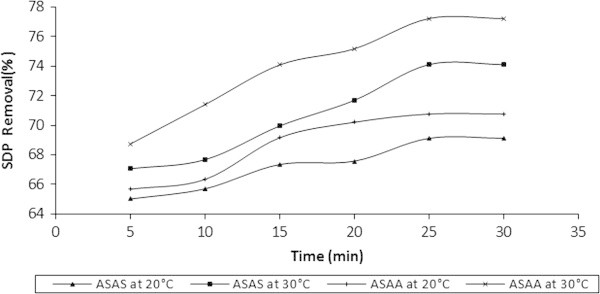
**Influence of temperature on the % removal of SDP from BRE onto ASAS and ASAA (adsorbent dose = 50 g/l, BRE volume = 20 ml, C**_**0**_ **= 161.92 mg/l, initial BRE pH, contact time = 30 min).**

**Figure 10:**
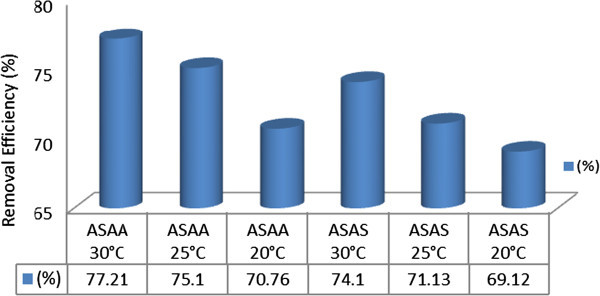
**Influence of temperature on removal efficiency of SDP on to ASAS and ASAA at contact time of 30 min (adsorbent dose = 50 g/l, BRE volume =20 ml, C**_**0**_ **= 161.92 mg/l, initial BRE pH).**

The removal of SDP from BRE onto ASAA and ASAS was investigated at following conditions: temperatures of 20, 25 and 30°C, 50 g/l adsorbent dose and contact time of 30 min. The effects, represented for only 20 and 30°C, are illustrated by the results in Figure 9. The Figure indicated that the percentage removal of SDP increased for ASAA and ASAS as the temperature of the system increased. Figure 10 shows the results obtained at 20, 25 and 30°C for the final SDP uptake from BRE onto ASAA and ASAS at 30 min of contact time. Quantitatively, at 20°C, 65.683 and 70.76% SDP removal were achieved for 5 and 30 min, respectively for ASAA (Figure 9). For ASAA at 30°C, 68.74 and 77.21% were obtained for 5 and 30 min, respectively. Results obtained for ASAS (Figure 9) indicated that 65.021 and 69.12% were recorded for 5 and 30 min, respectively at 20°C. At 30°C, ASAS recorded 67.02 and 74.100% for 5 and 30 min, respectively. Figure 10 indicated that when the temperature of BRE is placed at 20, 25 and 30°C, the ultimate achievable percentage of SDP removal in respect of ASAA, were 70.76, 75.10 and 77.21%, respectively. In respect of ASAS, the SDP removal achieved at 20, 25 and 30°C were 69.12, 71.13 and 74.10%, respectively.

Both Figures 9 and 10 indicated that the retention of SDP by the adsorbents increased while the temperature and time increased. The perceived increase of SDP uptake with temperature might be due to the acceleration of some originally slow adsorption steps or the creation of some active sites on the adsorbent surface (Hashem 2007; Nasssar and Magdy 1999). The enhanced mobility of SDP from the bulk solutions towards the adsorbent surface should also be taken into account (Hashem 2007; Yubin *et al.*^1998^). Increased temperature encouraged the process of agglomeration and widening adsorbent pore resulting in certain activation of the surface of the solid support (Larous *et al.*^2005^). Obtained results indicated clearly that the adsorption process under study was an endothermic process. This fact was illustrated in Section 3.6. Results similar to the one of this study had been reported by Khalid and Ahmad (1998).

### Influence of pH on the adsorptive removal of SDP from BRE

**Figure 11:**
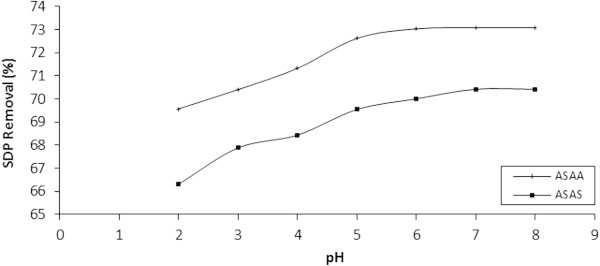
**Influence of pH on the adsorption of SDP from BRE on to ASAS and ASAA (adsorbent dose = 50 g/l, BRE volume = 20 ml, C**_**0**_ **= 161.92 mg/l, Temperature 30°C, contact time = 30 min).**

The pH of the aqueous solution was a vital controlling factor in the adsorption process and thus the impact of pH has been studied in the range of 2–8 as depicted graphically in Figure 11. As a general trend, the increase in pH, increased adsorption in a very determined sense, until a certain pH limit, beyond which the process became steady. This pH limit could be regarded as the optimal value.

In quantitative terms, the percentage SDP removal increased with pH from 2 to about 6.95 and remained unchanged thereafter. In respect of ASAA and ASAS, the SDP removal increased from 69.5768% at pH 2 to 73.0839% at pH 6.95 and 66.3161% at pH 2 to 70.4231% at pH 6.95, respectively. At pH greater than 6.95, the SDP removal remained constant till pH 8. The gentle decrease at about pH 3–6 of the initial rapid adsorption of SDP was presumed to be due to competitive adsorption between hydrogen ion and particles of the BRE. The adsorption at near neutral pH values could be attributed to the cellulose component of adsorbents where site binding adsorption might be occurring. This could be due to surface complex phenomena of functional groups present in the adsorbent (Hashem 2007; Menkiti et al. 2011a).

Graphical results of Figure 11 could be linked strongly to the influence of pH, in addition to the functional groups on the adsorbent and their ionic state at a particular pH (Mohan *et al.*^2007^; Genc et al. 2003). Equally, the apparent increment in adsorption with pH was believed to result from corresponding increase in the number of negatively charged sites. Consequently, the electrostatic attraction between the negative surface and the cationic BRE molecules increased with pH and reached saturation at about pH 6.95. Sivakumar and Palanisamy (2009) and Noroozi et al. (2007)) had reported similar results for the adsorption of basic red 29 onto Euphorbia antiquorum L and BR 41 onto silkworm pupa, respectively.

### Equilibrium isothermic analysis

The study of the adsorption isotherm is fundamental (Gräf et al. 2012), and played an important role in determination of the maximal capacity of adsorption, in addition to development of an equation which accurately represented the results that could be used for design purposes. Three equilibrium isotherms were analyzed: Langmuir, Freundlich and Temkin.

The Langmuir isotherm is arguably the best known of all isotherms describing adsorption. It applies to the cases of adsorption on completely homogenous surfaces where interactions between adsorbed molecules are negligible. The Freundlich isotherm is the earliest known relationship describing the adsorption isotherm. This empirical isotherm applies fairly well when describing the adsorption in dilute aqueous solution systems. Temkin isotherm applies on the bases that a fall in heat of sorption is linear rather than logarithmic as obtained in Freundlich equation. Heat of sorption of all molecules in the layer would decrease linearly with coverage due to sorbate- sorbent interactions (Larous *et al.*^2005^; Sujatha *et al.*^2008^).

The study under consideration for the equilibrium adsorption of SDP from BRE onto ASAA and ASAS were conducted at 20, 25 and 30°C. In order to evaluate the nature of the adsorption process, the obtained equilibrium data were fitted into Langmuir isotherm (Eq. Seven, Langmuir 1918); Freundlich isotherm (Eq. Eight, Freundlich 1906) and Temkin isotherm (Eq. Nine, Adouby *et al.*^2007^). Where applicable, both linear and non-linear mathematical expressions of the models and plots made are presented without details in Table 3.

**Table 3:** **Isotherm mathematical model equations used for adsorption data analysis**

Models	Non-linear form	Linear form	Plot made	Equation number
Langmuir				(7)
Freundlich				(8)
Temkin	-			(9)

The various isotherm parameters were determined from the corresponding slopes and intercepts. The level of accuracy of the models under study with experimental data was determined based on the squared linear regression coefficients (R^2^) and sum of the square error *SSE (%).* The higher is the value of R^2^ and the lower is the value of *SSE*; the better will be the goodness of fit. The expression for the evaluation of (*SSE%*) is presented as Eq. 10.10

**Figure 12:**
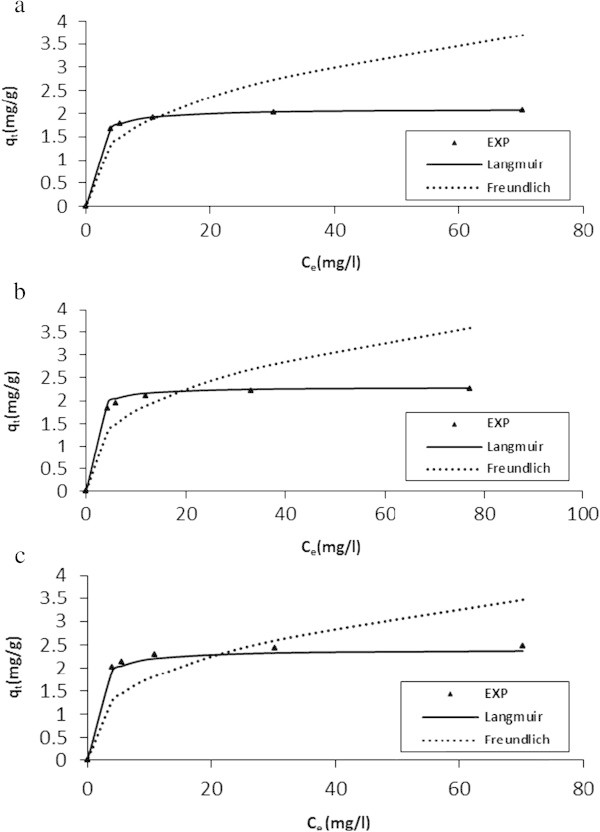
**Isotherm modeling at optimum conditons for the adsorption of SDP from BRE on to ASAA at: (a) 20°C, (b) 25°C, and (c) 30°C.**

**Figure 13:**
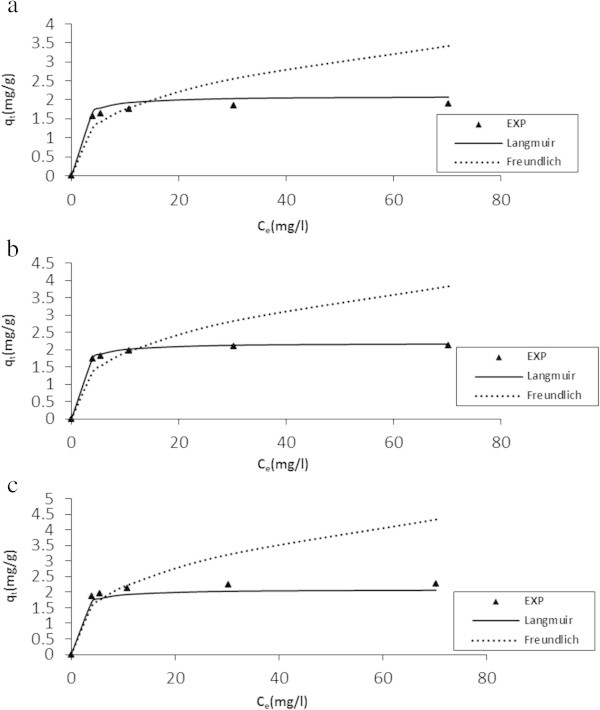
**Isotherm modeling at optimum conditons for the adsorption of SDP from BRE on to ASAS at: (a) 20°C, (b) 25°C, and (c) 30°C.**

The results of the Temkin, Freundlich and Langmuir constants and associated error calculations are presented in Table 4. Temkin isotherm with R^2^ < 0.480 has poor fit, thus no further consideration of the model was attempted. Hence, the equilibrium data obtained at 20°C, 25°C and 30°C have been shown (in the light of Langmuir and Freundlich Equations) as points, respectively, in Figure 12a to c for ASAA and Figure 13a to c for ASAS. The fit of the data (R^2^ > 0.99 and *SSE* < 0.10, Table 4) were found to be very well explained by the Langmuir isotherm. Thus, it could be generalized that the adsorption of SDP under the experimental conditions took place with the predominated Langmuir characteristics for the three temperatures under consideration. The adsorption capacity values, *q*_*m*_ for both ASAA and ASAS at the three temperatures were found to increase with increasing temperature. Similar trend was recorded for Langmuir constant K_L_ and Freundlich constant, K_f_ (ASAS). In respect of Langmuir isotherm, such a trend emphasized the homogeneity of the surface binding sites on the adsorbent (ASAA and ASAS) biomass towards BRE particles.

**Table 4:** **Isotherm parameters obtained from SDP adsorption on ASAA and ASAS at pH of 6.95, DA=50g/l, C**_**0**_ **= 161.92 mg/l**

Adsorbent	Temperature (°C)	Langmuir					Freudlich				Temkin			
		q_max_(mg/g)	K_L_(l/mg)	R^2^	R_L_	SSE (%)	K_F_(l/mg)	n	R^2^	SSE (%)	b_T_(KJ/mol)	K_T_(l/g)	R^2^	SSE (%)
ASAA	20	2.110	1.011	0.9916	0.0600	0.091	0.0810	2.801	0.893	1.041	7.221	13.381	0.2011	143.300
	25	2.300	1.039	0.9934	0.0059	0.096	0.807	2.914	0.891	2.213	6.483	450.969	0.3800	121.770
	30	2.500	1.060	0.9941	0.0570	0.093	0.800	2.900	0.896	1.881	4.017	5.891	0.1220	109.810
ASAS	20	2.100	1.001	0.9910	0.0061	0.093	0.790	2.900	0.839	1.371	6.121	13.314	0.1010	133.410
	25	2.150	1.020	0.9910	0.0060	0.098	0.830	2.780	0.771	2.710	3.874	17.967	0.4750	141.410
	30	2.300	1.050	0.9914	0.0584	0.096	0.950	2.780	0.799	2.011	2.212	170.331	0.1220	188.014

To confirm the favorability of the adsorption system, the dimensionless Hall separation factor, R_L_ was calculated by using Eq. 11 (Ruthsen 1984).11

*R*_*L*_ indicates the shape of the isotherm. This is: (i) unfavorable when *R*_*L*_ > 1, (ii) linear when *R*_*L*_ = 1 (iii) favorable when 0 < *R*_*L*_ < 1, and (iv) irreversible when *R*_*L*_ = 0. The calculated *R*_*L*_ values for the two adsorbents, shown in Table 4 were all in the range of 0–1, which confirmed that adsorption process was favorable within the studied experimental conditions.

In respect of Freundlich isotherm, favorability of the process is achieved if n lies in range of 1–10. Based on the values of Freundlich constant (n) displayed in Table 4, the present adsorption system could be considered favorable since n values lie between 1and 10 for all cases studied.

### Thermodynamic analyses of adsorption isotherm data

One way to elucidate the adsorption mechanism of SDP on the adsorbents was to calculate the thermodynamic parameters. These parameter estimates, evaluated the feasibility and exothermic nature of the adsorption process. Such parameters: standard free energy (*ΔG˚*), enthalpy (*ΔH˚*) and entropy change (*ΔS˚*) were obtained at 20, 25 and 30°C using binding Langmuir constant, *K*_*L*_ for the adsorption of SDP onto ASAA and ASAS. The three parameters were individually calculated using Eqs. 12–14.121314

The Gibbs free energy indicated the degree of spontaneity of the adsorption process and the higher negative values reflect more energetically favorable adsorption. The thermodynamic parameters evaluated for 20, 25 and 30°C are presented in Table 5. As shown, the negative values of *ΔG*˚ indicated the spontaneous nature of the adsorption process for both ASAA and ASAS. The positive values of *ΔH˚* indicated that the adsorption was endothermic while the positive values of *ΔS˚* pointed to an increased disorder of the system due to loss of the water which surrounded the BRE particles while adsorbing at the adsorbent (Suteu and Bilba 2005). Furthermore, it could be suggested that the driving force for adsorption was an entropic effect. This observation was reported by Wong et al. (2004) in earlier experimental results concerning organic adsorption mechanism.

**Table 5:** **Thermodynamic parameters for SDP on ASAA and ASAS**

Adsorbent	Temperature (°C)	ΔG (KJmol^−1^)	ΔH (KJmol^−1^)	ΔS (KJmol^−1^)
**ASAA**	20	−0.0266	↓	3.5196
	25	−0.0732	3.493	3.566
	30	−0.1467	↑	3.6397
**ASAS**	20	−0.0266	↓	2.5037
	25	−0.0491	2.4771	2.5262
	30	−0.1229	↑	2.6000

Table 5 shows that *ΔG˚* values were increasingly negative with temperature, which indicated the increasing feasibility and spontaneity of the adsorption process. The adsorption of SDP onto ASAA was greater than that of SDP onto ASAS overall. Also, from Table 5, the values of *ΔS*˚ are increasingly positive with temperature, an indication of increasing randomness of solid–liquid interface with increasing temperature (Debnath and Ghosh 2008). From the table, the values of *ΔH*^°^ recorded for ASAA and ASAS are 3.493 kJ/mole and 2.477 kJ/mole, respectively. The values are found to be less than 40 kJ/mole which indicated that physisorption dominated the adsorption of SDP onto ASAA and ASAS. The present results are found to follow similar trend with the results reported by Patel and Suresh (2008) on the biosorption of reactive black 5 dyes by *Aspergillus foetidus*.

### Surface packing, surface charge and hopping number

To further account for the adsorption behavior of the SDP onto acid treated (ASAA) and salt treated (ASAS) biomasses as a result of surface modification, the packing of SDP on the biomass surface was evaluated. This involved thermodynamic analysis of adsorption potential, adsorption density at a fixed temperature (30°C) and initial adsorbate concentration (161.920 mg/l). The adsorption potential, *A* and the adsorption density, Γ, were obtained by calculation using Eqs. 15 and 16, respectively (Horsfall and Spiff 2005).1516

where: *C*_*o*_ and *C*_*e*_ are initial and equilibrium concentrations in mg/cm^3^, Γ is the adsorption density in mg/m^2^, *Z = 4* is the valency of organic carbon, which is a major constitutent (Driessen and Vereijken 2003; Kanagachandran and Jayaratne 2006; Janhom et al. 2009; Menkiti 2010) of SDP in the BRE, *r* = 0.70 ×10^- 9^ m is the effective radius of the organic carbon enriched-SDP reported elsewhere (Crystalmarker 2014; United States Environmental Protection Agency USEPA 1999), *R* is gas constant, and *T* is the absolute temperature (K).

**Figure 14:**
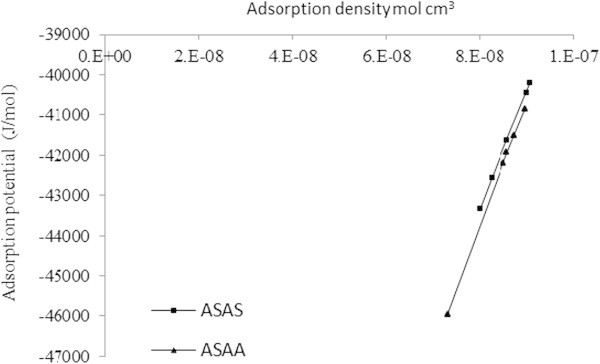
**Adsorption potential vs. adsorption density.**

The graphical representations are depicted in Figure 14. The adsorption potential for both ASAA and ASAS increased with increment in adsorption density. However, adsorption potential values for acid treated adsorbent (ASAA) were higher than the salt treated biomass (ASAS). The higher uptake of SDP observed for ASAA was primarily due to the association of phosphoric acid group. Acid treatment significantly enhanced the adsorption potential of the biomass, which meant an increased adsorption density on the ASAA.

Adsorption behavior arising from the adsorbent modification could be viewed from the aspect of vacant sites. The probability of finding vacant sites on the adsorbent surface was correlated by number of hopping (n) done by the BRE particles before sticking on an adsorption site. The expressions relating the number of hopping (n) and that of surface coverage are given in Eqs. 17 and 18 (Higachi *et al.*^1984^; Horsfall and Spiff 2005).1718

**Figure 15:**
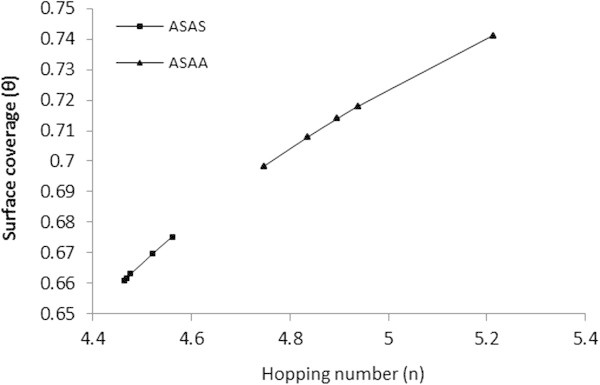
**Surface coverage vs. Hopping number (n).**

Plot of surface coverage *(θ)* against hopping number (*n*) was made and depicted in Figure 15. The figure indicates that the migration of SDP to the vacant sites on the adsorbent surface increased with increase in surface coverage. The smaller the hopping number, the faster adsorption occurred. The difference observed in *θ* and *n* for the ASAA and ASAS (Figure 14) indicated the ease with which ASAA found binding site when compared with ASAS. This could attributed to the association of phosphoric acid group in ASAA.

### Adsorption kinetic analysis

Kinetic and equilibrium data are pertinent in the evaluation of adsorption dynamics and by extension, the optimization of residence time for the uptake of SDP on ASAA and ASAS. Adsorption kinetics could be controlled by several independent processes (bulk diffusion, film diffusion, chemical reaction, intra-particle diffusion, temperature, pH, etc.) that could act in series or in parallel (Abia and Asuquo 2006). In order to investigate the kinetic of BRE adsorption on ASAA and ASAS, the data obtained at 30°C have been analyzed by pseudo first order(PFO) (Langergren 1898, Eq. Nineteen), pseudo second order(PSO) (Ho and Mckays 1999, Eq. Twenty); Elovich (Chien and Clayton 1980, Eq. Twenty-one) and Bhattacharya – Venkobachar (BVM) (Bhattacharya and Venkobachar 1984, Equation Twenty-two) kinetic model equations and subsequently subjected to accuracy tests via R^2^ and *SSE* (Eq. 6).

The mathematical linear forms of the equations used and the plots made for analyzing the data have been given without details in Table 6. The associated kinetic parameters have been determined from the intercepts and slopes of the respective linear plots (graphs omitted) of the kinetic equations. The evaluated kinetic parameters are shown in Table 7. The results indicated that among the four kinetic models evaluated, pseudo second order model generated the best fit with the adsorption kinetic data of the investigated system. All the correlation coefficients obtained were greater than 0.99. The pseudo first order equation showed next good fit with the adsorption data, followed by the Elovich model. Lastly, Bhattacharya–Venkobachar (BVM) with R^2^ **<** 0 depicted the worst fit. It could be presumed from the present data, that ASAA kinetically had a better result than ASAS in respect of pseudo first order and pseudo second order.

**Table 6:** **The kinetic equations used for analysis of kinetic data**

Kinetic equation	Linear form	Plot made	Equation number
PFO	ln(*q*_*e*_ − *q*_*t*_) = ln *q*_*e*_ − *K*_1_ *t*	ln(*q*_*e*_ − *q*_*t*_) *vs. Time*	(19)
PSO			(20)
Elovich		*q*_*t*_ *vs. Time*	(21)
BVM	ln[1 − (*U*)*T*] = *K*_*B*_ *t*	ln[1 − (*U*)*T*] *vs. Time*	(22)

**Table 7:** **Kinetic parameters of the adsorption process at 30°C ,pH 6.95, D**_**A**_ **= 50 g/l , C**_**0**_ **= 161.92 mg/l**

Kinetic model	Parameter	Adsorbent variant	
		ASAA	ASAS
PFO	K_1_(/min)	0.0218	0.0424
	q_e_	0.7044	0.6017
	R^2^	0.9968	0.9624
	SSE(%)	0.1542	0.1550
PSO	K_2_(g/mg.min)	0.2809	0.3462
	q_e_(mg/g)	1.6920	1.6233
	R^2^	0.9986	0.9984
	SSE(%)	0.1477	0.1473
Elovich	α (mg/g.min)	39.4302	102.4312
	β (mg/g.min)	5.5309	6.4184
	R^2^	0.9895	0.9752
	SSE(%)	0.3011	0.3101
BVM	K_B_(/min)	−0.0922	−0.0681
	R^2^	−0.7053	−4.4455
	SSE(%)	163.1241	169.3247

## Conclusion

African star apple shell biomass (ASAA and ASAS) was able to adsorb SDP from brewery effluent (BRE). The removal of SDP from BRE using ASAA and ASAS was a function of contact time, bioadsorbent dosage, temperature and pH. Adsorption capacity increased with increasing temperature. The optimum SDP removals were achieved at pH 6.95, 50 g/l dose and 30°C. Among the isotherm models considered, equilibrium data fitted the Langmuir model best within the studied experimental conditions. The kinetic data agreed very well with the pseudo-second order equation. The calculated thermodynamics parameters showed that the related adsorption systems were favorable, endothermic and spontaneous for both ASAA and ASAS.
